# Combined Blockade of GARP:TGF-β1 and PD-1 Increases Infiltration of T Cells and Density of Pericyte-Covered GARP^+^ Blood Vessels in Mouse MC38 Tumors

**DOI:** 10.3389/fimmu.2021.704050

**Published:** 2021-07-27

**Authors:** Charlotte Bertrand, Pierre Van Meerbeeck, Grégoire de Streel, Noora Vaherto-Bleeckx, Fatima Benhaddi, Loïc Rouaud, Agnès Noël, Pierre G. Coulie, Nicolas van Baren, Sophie Lucas

**Affiliations:** ^1^de Duve Institute, Université Catholique de Louvain, Brussels, Belgium; ^2^GIGA-Cancer Research Center, University of Liège, Liège, Belgium; ^3^Walloon Excellence in Life Sciences and Biotechnology (WELBIO), Wavre, Belgium

**Keywords:** GARP, TGF-β1, cancer immunotherapy, immunosuppression, immune checkpoint inhibition

## Abstract

When combined with anti-PD-1, monoclonal antibodies (mAbs) against GARP:TGF-β1 complexes induced more frequent immune-mediated rejections of CT26 and MC38 murine tumors than anti-PD-1 alone. In both types of tumors, the activity of anti-GARP:TGF-β1 mAbs resulted from blocking active TGF-β1 production and immunosuppression by GARP-expressing regulatory T cells. In CT26 tumors, combined GARP:TGF-β1/PD-1 blockade did not augment the infiltration of T cells, but did increase the effector functions of already present anti-tumor T cells. Here we show that, in contrast, in MC38, combined GARP:TGF-β1/PD-1 blockade increased infiltration of T cells, as a result of increased extravasation of T cells from blood vessels. Unexpectedly, combined GARP:TGF-β1/PD-1 blockade also increased the density of GARP^+^ blood vessels covered by pericytes in MC38, but not in CT26 tumors. This appears to occur because anti-GARP:TGF-β1, by blocking TGF-β1 signals, favors the proliferation of and expression of adhesion molecules such as E-selectin by blood endothelial cells. The resulting densification of intratumoral blood vasculature probably contributes to increased T cell infiltration and to the therapeutic efficacy of GARP:TGF-β1/PD-1 blockade in MC38. We conclude from these distinct observations in MC38 and CT26, that the combined blockades of GARP:TGF-β1 and PD-1 can exert anti-tumor activity *via* multiple mechanisms, including the densification and normalization of intratumoral blood vasculature, the increase of T cell infiltration into the tumor and the increase of the effector functions of intratumoral tumor-specific T cells. This may prove important for the selection of cancer patients who could benefit from combined GARP:TGF-β1/PD-1 blockade in the clinics.

## Introduction

TGF-β1 is a potent immunosuppressive cytokine produced by most cells in an inactive, latent form. In its latent form, the mature TGF-β1 dimer is non-covalently associated to the Latency Associated Peptide (LAP), which prevents receptor binding and signaling by the cytokine. Some cell types can activate latent TGF-β1 in response to various stimuli by releasing mature TGF-β1 from LAP, *via* cell-type specific mechanisms. Regulatory T cells (Tregs) stimulated through the T cell receptor (TCR) produce latent TGF-β1 covalently linked to transmembrane protein GARP. Presentation of GARP:(latent)TGF-β1 complexes, and the interaction with integrin αVβ8 on the surface of TCR-stimulated Tregs, leads to TGF-β1 activation and autocrine or short-distance paracrine TGF-β1 activity (1–4). In cancer patients, Tregs can exert detrimental immunosuppression and thus limit the efficacy of immunotherapy (5, 6).

We previously developed monoclonal antibodies (mAbs) that bind GARP:TGF-β1 complexes and block TGF-β1 activation and immunosuppression by human or mouse Tregs (4, 7, 8). Anti-GARP:TGF-β1 mAbs inhibited the immunosuppressive function of human Tregs in a xenogeneic model of graft-versus-host disease induced by the transfer of human PBMCs in NSG mice (7). More recently, we reported that anti-GARP:TGF-β1 combined with anti-PD-1 induced immune-mediated rejections of CT26 and MC38 tumors resistant to anti-PD-1 alone (8). Blocking TGF-β1 activation by GARP-expressing Tregs was sufficient for anti-GARP:TGF-β1 to overcome resistance to anti-PD-1 in these tumor models. Indeed, the anti-tumor activity of combined GARP:TGF-β1/PD-1 blockade: i) occurred without Treg depletion, ii) was observed using anti-GARP:TGF-β1 incapable of binding Fcγ receptors, and iii) was lost in MC38 tumor-bearing mice carrying a Treg-specific deletion of the *Garp* gene (8). In contrast, blocking TGF-β1 activation by GARP-expressing platelets was not required, as anti-tumor activity of combined GARP:TGF-β1/PD-1 blockade was conserved in MC38 tumor-bearing mice carrying a platelet-specific deletion of the *Garp* gene. Thus, in MC38, the predominant source of active TGF-β1 that needs to be blocked by anti-GARP:TGF-β1 to overcome resistance to anti-PD-1 are Tregs, but not platelets (8).

Further characterizing its mode of action, we observed that combined GARP:TGF-β1/PD-1 blockade increased the effector functions of anti-tumor CD8 T cells already present within CT26 tumors, without augmenting the immune cell infiltration (8). Together with our observation that GARP-expressing Tregs are found mostly in human melanoma metastases that are already infiltrated by activated T cells, this led us to suggest testing anti-GARP:TGF-β1 to overcome resistance to PD-1/PD-L1 blockade in patients with inflamed tumors.

Different modes of action were proposed to explain the anti-tumor activity of PD-1/PD-L1 blockade combined with mAbs that block latent TGF-β1 activation (9) or neutralize all three TGF-β1, β2 and β3 isoforms (10, 11). In these reports, infiltration of immune cells in tumors was increased as a consequence of either increased CD8 T cell entry from blood vessels (9), or reduced TGF-β1 signaling in stromal fibroblasts with subsequent increased CD8 T cell penetration towards the center of tumors (10, 12). The variations in the mode of action could result from the different tumor models that were used in our laboratories, or from the different cellular sources of TGF-β activity that were blocked by the mAbs. While anti-GARP:TGF-β1 selectively blocks TGF-β1 released from GARP-expressing cells, other mAbs block TGF-β1 activation, or neutralize the activity of all three TGF-β isoforms, whatever their cellular source. It is noteworthy that in addition to Tregs, murine and human endothelial cells and platelets do also express surface GARP:TGF-β1 complexes. Anti-GARP:TGF-β1 mAbs could thus exert anti-tumor activities *via* multiple modes of action, by directly or indirectly targeting different cell types depending on the tumor microenvironment (2, 8, 13).

We thus resorted to determine if the anti-tumor activity of combined GARP:TGF-β1/PD-1 blockade could encompass other mechanisms than increasing the effector functions of anti-tumor CD8 T cells already present within tumors, as observed in CT26.

## Materials and Methods

### Mice

BALB/c and C57BL/6 mice were bred at the SPF animal facility of the UCLouvain. The facility is controlled to maintain the temperature between 20 and 24°C; humidity rate between 40 and 65% and day–night cycles of 12 h–12 h. All animal studies were performed in accordance with national and institutional guidelines for animal care, under permit numbers 2015/UCL/MD/19 and 2019/UCL/MD/032 at the UCLouvain.

### Antibodies

Anti-mouse GARP:TGF-β1 clone 58A2 was described previously (8). Batches of 58A2 under mIgG1 or mIgG2a DANA formats, and corresponding isotype controls (motavizumab) were kindly provided by Dr. Bas van der Woning (argenx BV). mIgG1 and mIgG2a DANA antibodies do not exert FcγR-dependent activities (8, 14). Anti-PD-1 clone RMP1-14 (mIgG2a FcSilent format) was purchased from Absolute Antibodies. Anti-active TGF-β1,2,3 clone 1D11 was purchased at BioXcell.

### Cells

CT26 and MC38 colon carcinoma cell lines were maintained *in vitro* as a monolayer culture in Iscove’s Modified Dulbecco Medium (CT26), or Roswell Park Memorial Institute Medium (MC38), supplemented with 10% fetal calf serum (FCS) and 10 mM Hepes, 1 mM sodium pyruvate and 0.1 mM non-essential amino acids, at 37°C in an atmosphere of 8% (CT26) or 5% (MC38) CO_2_ in air. Murine tumor cells in exponential growth phase were harvested, washed in PBS, and resuspended in endotoxin-free Dulbecco’s PBS (Millipore) prior to inoculation into mice.

Endothelial cell lines C166 and MS1 were purchased at ATCC or kindly provided by the group of Agnès Noël (ULiège, Belgium), respectively. Both were maintained *in vitro* as a monolayer culture in Dulbecco’s Modified Eagle Medium supplemented with 10% FCS, at 37°C in an atmosphere of 5% CO_2_ in air.

### Animal Experiments

On day 0, live CT26 cells (10^6^ cells/mouse) or MC38 cells (1 × 10^6^/mouse for mIF experiments or 1.5 × 10^6^ cells/mouse for flow cytometry experiments) were injected *s.c.* into 7- to 8-week-old syngeneic mice. Large (D) and small (d) tumor diameters were measured with a caliper every 2 or 3 days starting on day 6. Tumor volumes were calculated as follows: V = π × D × d^2^/6. On days indicated in the figure legends, mice received intraperitoneal (*i.p.*) injections of the following mAbs (250 µg of each), administered alone or combined as indicated in the figure legends: isotype control (motavizumab), anti-GARP:TGF-β1 (clone 58A2) and/or anti-PD-1 (clone RMP1-14 FcSilent). Anti-GARP:TGF-β1 injected in BALB/c or C57BL/6 mice were under mIgG2a DANA or mIgG1 formats, respectively (corresponding isotypes were used for the motavizumab control). All mice were sacrificed on day 13 or 14, and tumors were collected after sacrifice for further analyses.

### Multiplexed Immunofluorescence on Mouse Tumor Sections

Immediately after collection, CT26 and MC38 tumors were fixed in formaldehyde (FA) 4% during 24 h, then embedded in paraffin using the Tissue-Tek VIP (Sakura). Formaldehyde-fixed paraffin-embedded (FFPE) tumors were cut in 5 µm-thick sections and mounted on a microscope slide. Slides were deparaffinized in subsequent baths of HistoClear and decreasing concentrations of ethanol. Antigen retrieval was performed in Tris–EDTA buffer (pH 9) with microwave treatment. Endogenous peroxidases were inactivated using a peroxidase block reagent (Enzo) for 15 min and sections were permeabilized and blocked with Tris Buffered Saline (TBS) supplemented with 0.1% Tween 20, 2% milk, 5% Bovine Serum Albumin (BSA), and 1% human Ig for 30 min. The following primary antibodies were used (incubation time was 90 min) for immunofluorescent staining on these sections, alone or in combination, as indicated in the figure legends: anti-CD3 (Abcam, clone SP7, diluted 1:500), anti-CD8 (Cell signaling, clone D4W2Z, diluted 1:400), anti-CD146 (Abcam, clone EPR3208, diluted 1:250), anti-PDGFRβ (Abcam, clone Y92, diluted 1:200). Ready-to-use EnVision+ System-HRP Labelled Polymer Anti-Rabbit (Dako) was used (60 min) as a secondary antibody for all antibodies cited above, followed by incubation with a Tyramide Reagent coupled either to Alexa Fluor 488, Alexa Fluor 555 or Alexa Fluor 647 (Thermo, diluted 1:200), prepared in a buffer containing 0.1 M boric acid, 3 M NaCl, 0.1% Tween 20 (pH 7.8) supplemented with 0.003% H_2_O_2_, for 10 min. After each staining, an additional step of heat-mediated antibody elution in citrate buffer (pH 6) was performed in a microwave oven. Counterstaining of nuclei was performed with Hoechst33258 reagent (1:1,000) for 5 min. Slides were mounted with Fluorescent Mounting Medium (Dako) and covered.

Another piece of tumor was frozen, embedded in OCT and cut in 7 µm-thick sections to perform staining, including GARP. Cryosections were fixed for 5 min in FA 4%. Endogenous peroxidase blocking and permeabilization were performed, as described above. For GARP staining, clone YGIC86 (Thermo, diluted 1:80) was used as the primary antibody and the signal was amplified with ready-to-use ImPRESS HRP Goat Anti-Rat IgG (Vector) and a Tyramide Reagent, coupled to Alexa Fluor 555. Slides were counterstained and mounted as described previously.

Digital 3 or 4-color images of the stained tissue sections were acquired with a Pannoramic P250 Flash III scanner (3DHistech) equipped with a Plan-Apochromat 20×/N.A. 0.8× objective (Carl Zeiss) and with a Point Grey Grasshopper 5MP camera, using DAPI1, FITC, SpRed and Cy5 filter sets (Semrock).

### Immunofluorescence Image Analysis

mIF images were analyzed with the Halo software (Indicalabs). The Cytonuclear FL module was used for the quantification of T cell densities as well as for the calculation of distances between the T cells to the tumor periphery (Dp), and to nearest neighbor endothelial cell. To calculate distances, we designed a dedicated R script that uses the position and phenotype of each event identified by Halo. For **Figure 2**, the tumor periphery was defined using the cells that are at the extremities of the tissue section, and the tumor center was calculated as centroid. A mean radius length was calculated for each section based on the lengths of all radiuses measured in the section (one radius represents the distance between the centroid and one DAPI^+^ nucleus located on the tumor periphery). Five CT26 tumors were excluded from this analysis because their centroid were located outside of the tissue, due to their moon-like shape. The distance of each CD8 T cell to their closest endothelial cell was calculated using the nearest neighbor analysis. For quantification of blood vessels, the Object Quantification FL module of Halo was used, with each object corresponding to an individual blood vessel.

### Flow Cytometry Analyses

On the day indicated in the figure legends, tumors were harvested and mechanically dissociated in the presence of enzymes (Collagenase I 100 mg/ml, Life Tech; Collagenase II 100 mg/ml, Life Tech; Dispase 1 mg/ml, Life Tech; and DNAse I 0.4 U/ml, Roche), using two cycles in the GentleMacs disruptor (Miltenyi) separated by 30 min of incubation at 37°C under continuous agitation. Tumor cell homogenates were clarified through 70 and 40 µm filters. Single cell suspensions were counted on a Luna^®^ cell counter with a live-dead cell marker, then pelleted and resuspended in PBS containing 2 mM EDTA and 1% FCS for immediate staining, or in X-Vivo 10 medium (Invitrogen) to shortly incubate cells prior to staining.

Cells used for immediate staining were incubated with antibodies against surface markers (CD45, clone 30F-11; CD4, clone RM4-5; CD8a, clone 53-6.7) (Biolegend) in the presence of a viability dye (eBioscience) and anti-CD16/32 to block FcγRs. To identify MC38 tumor-specific T cells, cells were also incubated with an H2-K^b^-p15E (KSPWFTTL) pentamer coupled to APC (ProImmune). One million cells per tumor were kept for *ex vivo* incubation with Brefeldin A (Sigma, 5 µg/ml) to increase the accumulation of cytokines in the Golgi/endoplasmic reticulum. No stimulation reagents were applied. Anti-CD107a mAb coupled to BV421 (clone 1D4B, Biolegend) was added to the mix. After 4 h of incubation at 37°C, cells were stained with antibodies against surface markers (CD45, CD4, CD8a) (Biolegend) in the presence of a viability dye (eBioscience) and an anti-CD16/32, fixed and permeabilized with the Cytofix/Cytoperm kit (BD Biosciences), then stained with antibodies against intracellular cytokines (IFNγ, clone XMG1.2; TNFα, clone MP6-XT22) (Biolegend) in the presence of additional anti-CD16/32.

C166 and MS1 cells were incubated with anti-CD16/32 antibody then stained with a biotinylated anti-GARP:TGF-β1 antibody (clone 58A2) and a streptavidin coupled to PE. Analyses were performed on a FACS LSRFortessa flow cytometer (DIVA, BD Biosciences) and data were computed using the FlowJo software (Tree Star).

### RT-qPCR of Mouse Tissue Samples and Cell Lines

Mouse tumor fragments were collected and stored at −80°C until processing. After tissue disruption with the TissueLyser (Qiagen), total RNA was extracted using NucleoSpin Mini Columns (Macherey Nagel). RNA was reverse transcribed with Maxima First Strand cDNA Synthesis Kit (Thermofisher). For **Figure 6**, the MS1 cell line and splenocytes obtained from BALB/c mice were treated with recombinant TGF-β (rTGF-β) at 1 ng/ml for 24 h, then harvested and pelleted. RNA extraction and reverse transcription were performed as described above. qPCR were performed in the QuantStudio3 device (Thermofisher) in reaction volumes of 20 µl containing 0.025 U/µl of Takyon Master Mix (Eurogentec), 300 nM of each primers, 100 nM of Takyon probe, under either standard conditions (95°C for 3′; 45 cycles of 95°C for 10″ and 60°C for 30″) or fast conditions (95°C for 3′; 95°C for 3″ and 60°C for 30″) depending on amplicon size. The sequences of primers and probes are listed in **Table S1**.

### Thymidine Incorporation Assay

C166 and MS1 endothelial cells (2,000 cells/well) were incubated in the presence of rTGF-β1, for the duration indicated in the figure legends. One µCurie of [^3^H]-thymidine (^3^H-T) was added during the last 24 h of culture. On the day of revelation, cells were incubated with trypsin and aspirated on a 96-filter plate (Unifilter GF/C) using a harvester (Packard Filtermate 196). The plate was washed several times with water. Radioactivity was measured by a scintillation counter (Packard Microplate Scintillation Counter), which calculates the counts per minute (cpm).

### Statistical Analyses

All statistical analyses were performed using the JMP^®^Pro 15 software. Comparisons of measurements taken at a single time point were performed using a two-tailed, non-paired, non-parametric Wilcoxon test. *Post-hoc* Tukey’s test was performed to adjust for multiple comparisons. The number of experiments and the number of mice (n) in the various experimental groups are indicated in the corresponding figure legend.

## Results

### Combined GARP:TGF-β1/PD-1 Blockade Increases Infiltration of T Cells in MC38 but Not in CT26 Tumors

To compare the mechanism of action of combined GARP:TGF-β1/PD-1 blockade in two mouse tumor models, we injected MC38 or CT26 cells subcutaneously (*s.c.*) in syngeneic C57BL/6 or BALB/c mice, respectively. We then started intraperitoneal (*i.p.*) administrations of either isotype control, anti-GARP:TGF-β1 and/or anti-PD-1 mAbs after 6 days, when the tumors were well established in all mice. A total of three injections of single or combined mAbs were administered on days 6, 9 and 12, and tumors were collected on day 13. This time point was chosen based on our previous study, which showed that similar administration regimens significantly increased the frequency of complete, immune-mediated rejections of MC38 and CT26 tumors in mice treated with anti-GARP:TGF-β1 + anti-PD-1, as compared with anti-PD-1 alone. Notably, rejections occurred between days 15 and 40 in both MC38- and CT26-tumor bearing mice, leaving no sufficient tumor material for analyses after day 13 (8). Here on day 13, and as expected, volume and weight of tumors were already significantly reduced in mice treated with anti-GARP:TGF-β1 + anti-PD-1, or anti-PD-1 alone, as compared with controls (**Figure S1**).

**Figure 1:**
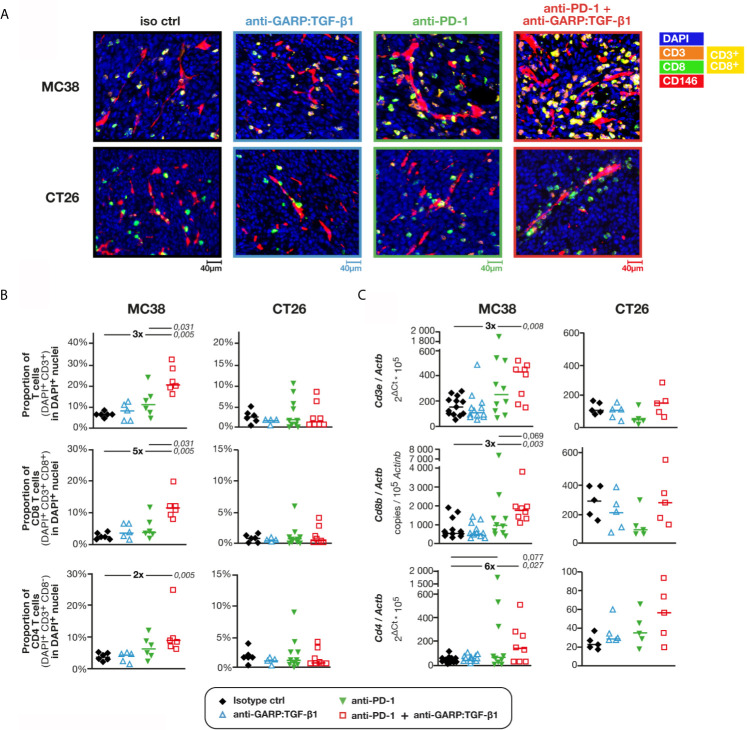
Combined GARP:TGF-β1/PD-1 blockade increases infiltration of total, CD8 and CD4 T cells in MC38, but not in CT26 tumors. C57BL/6 or BALB/c mice were injected on day 0 with live MC38 or CT26 cells, respectively, and treated on days 6, 9 and 12 with anti-GARP:TGF-β1 (clone 58A2 mIgG1 in C57BL/6 mice; clone 58A2 mIgG2a DANA in BALB/c mice), anti-PD-1 (clone RMP1-14 mIgG2a FcSilent), a combination of both mAbs or the corresponding isotype controls. Tumors were collected on day 13 and fragments were analyzed by mIF and quantitative digital imaging or RT-qPCR. **(A)** Representative images of FFPE tumor sections stained with anti-CD3 antibody (orange), anti-CD8 antibody (green), anti-CD146 antibody (red) and Hoescht (DAPI, blue). CD8 T cells (CD3^+^CD8^+^) appear in yellow. **(B)** Proportions (%) of total, CD8 and CD4 T cells in DAPI^+^ nuclei measured with Indicalabs Halo software. Results obtained in one experiment for MC38 (n = 5–6 mice/group) and pooled from two independent experiments for CT26 (n = 3–6 mice/group in each experiment). One tumor section analyzed per mouse. **(C)** Expression levels of *Cd3e*, *Cd8* and *Cd4* mRNA relative to housekeeping gene *Actb* in MC38 and CT26 samples. Samples from two independent experiments for MC38 (n = 4–7 mice/group in each experiment) and one experiment for CT26 (n = 5/group). Samples from the second MC38 experiment shown in c were also analyzed in **Figure S2** by flow cytometry. Data points represent values in individual mice. Horizontal bars: median per group. P values <0.05, calculated with a two-sided Wilcoxon test, are indicated in italics. Numbers in bold: fold-change between the indicated groups.

We used digitalized multiplex immunofluorescence stainings (mIF) of formaldehyde-fixed paraffin-embedded (FFPE) sections to quantify T cell infiltration in the tumors (**Figures 1A, B**). In tumors from untreated mice, the proportion of CD3^+^ cells among DAPI^+^ nuclei was approximately 3-fold higher in MC38 (6.8% ± 0.4%; median ± interquartile range, or IQR) as compared with CT26 tumors (2.3% ± 1.4%) (**Figure 1B**). Consistent with our previous observations, administration of anti-GARP:TGF-β1 or anti-PD-1 alone did not significantly increase T cell infiltration in MC38 or CT26 tumors, and the anti-GARP:TGF-β1 + anti-PD-1 combination did not increase T cell infiltration in CT26 [**Figure 1B** and (8)]. However, the combination caused a significant 3-fold increase of the proportion of T cells infiltrating MC38 tumors in mice treated with the anti-GARP:TGF-β1 + anti-PD-1 combination (20.9% ± 7.5%) (**Figure 1B**). T cell infiltration was significantly higher with this combination than with the anti-PD-1 alone. Both CD8 and CD4 T cells contributed to the infiltration, but CD8 T cells more so (≈5-fold) than CD4 (≈2-fold). RT-qPCR for genes *Cd3e*, *Cd8b* and *Cd4* confirmed increased infiltration of total, CD8 and CD4 T cells in MC38 tumors after combined GARP:TGF-β1/PD-1 blockade (**Figure 1C**).

We observed with flow cytometry that the proportion of CD8 and CD4 T cells among MC38-infiltrating leukocytes (CD45^+^ cells) were not modified after GARP:TGF-β1/PD-1 blockade, indicating that the augmented T cell infiltration resulted from an increased infiltration of total leukocytes and not of a particular cell subset (**Figure S2A**). Lymphocytes directed against the tumor-specific antigen p15E were identified by staining with a fluorescent H2-K^b^ pentamer loaded with the p15E peptide (15). Their proportions among leukocytes (7.6% ± 4.9%) or CD8 T cells (19.1% ± 11.6%) did not differ between the untreated or treated MC38 tumors (**Figure S2A**). We also did not find differences between the proportions of CD8 T cells or tumor-specific CD8 T cells that produced IFNγ and/or TNFα, or expressed the surface marker of degranulation CD107a (**Figures S2B**). The only difference was the proportions of CD4 T cells producing IFNγ, increased after combined GARP:TGF-β1/PD-1 blockade as compared with PD-1 blockade alone or controls (**Figure S2B**). RT-qPCR analyses confirmed these observations (**Figure S2C**).

We conclude that in MC38 tumors, the combined GARP:TGF-β1/PD-1 blockade increases the infiltration of T cells, including activated tumor-specific CD8 T cells. This contrasts with our previously published observations in CT26, where combined GARP:TGF-β1/PD-1 blockade did not increase T cell infiltration, but did augment the effector functions of the anti-tumor T cells that were already present in the tumors (8).

### Combined GARP:TGF-β1/PD-1 Blockade Does Not Increase the Proliferation or Penetration of Tumor-Infiltrating CD8 T Cells in MC38 or CT26 Tumors

The increased T cell infiltration into MC38 following GARP:TGF-β1/PD-1 blockade could result from a local T cell proliferation within MC38, but not CT26 tumors. However, we observed no change in levels of Ki-67 in CD8 T cells or in the proportion of proliferating Ki-67^+^ cells among CD8 T cells in any treatment group in MC38 or CT26 tumors (**Figures S3A, B**).

**Figure 2:**
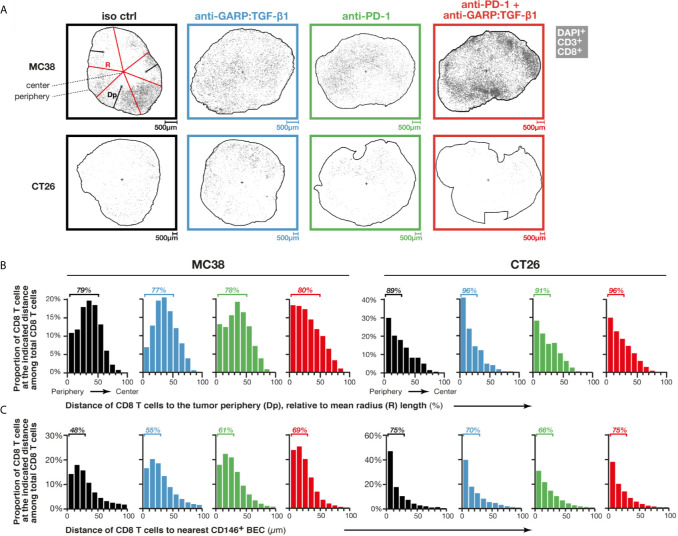
Combined GARP:TGF-β1/PD-1 blockade induces entry of T cells *via* intratumoral vessels rather than penetration from the tumor periphery. Sections of FFPE tumors from **Figures 1A, B** analyzed by mIF and quantitative digital imaging. Treatment groups are indicated in colored font on top (black: isotype control; blue: anti-GARP:TGF-β1; green: anti-PD-1; red: anti-PD-1 + anti-GARP:TGF-β1). **(A)** Digital representations generated by the Halo software of one representative tumor section per treatment group, with CD8 T cells (DAPI^+^CD3^+^CD8^+^) indicated as grey dots. The outer edge of the tumors (periphery) is delineated by a black line, and the position of the tumor center, calculated as a centroid, is depicted by a black cross. On one representative section (top left), the distance to the periphery (Dp) of a few CD8 T cells are illustrated by black lines, and a few radiuses (R) are depicted by red lines. A mean radius length was calculated for each section based on the lengths of all radiuses measured in the section (one radius represents the distance between the centroid and one DAPI^+^ nucleus located on the tumor periphery). **(B)** Distribution of Dp of CD8 T cells relative to mean radius length, *i.e.* proportion of CD8 T cells within all CD8 T cells in a given tumor section that are located at the indicated relative Dp (0–10%, 10–20%,…). Bars represent mean proportions in all sections analyzed in one treatment group. **(C)** Distribution of CD8 T cell distances to the nearest endothelial cell (DAPI^+^CD146^+^), as determined using nearest neighbor analysis. Bars represent mean proportions of CD8 T cells within all CD8 T cells in a given section that are located at the indicated distance (0–10 µm, 10–20 µm,…) from the nearest blood endothelial cell (BEC). Results from one experiment for MC38 (n = 5–6 mice/group) and pooled from two independent experiments for CT26 (n = 3–6 mice/group in each experiment). One tumor section analyzed per mouse.

Increased T cell infiltration could also result from an increased penetration of T cells from the periphery towards the center of MC38 tumors, as shown in EMT6 treated with a combination of anti-active TGF-β1, β2 and β3 (clone 1D11) and anti-PD-L1 (clone 6E11) (10). We measured the distance separating each CD8 T cell from the closest point on the tumor periphery in MC38 and CT26 FFPE sections, stained by mIF (**Figure 2A**), then examined the distribution of all CD8 T cells at various distances from the periphery relative to mean radius length (**Figure 2B**). In control MC38 tumors, the vast majority of CD8 T cells were closer to the tumor periphery than its center (79% of CD8 T cells at <50% mean radius length). Treatment with anti-GARP:TGF-β1, combined or not with anti-PD-1, did not modify this distribution (**Figure 2B**). In control CT26, a majority of CD8 T cells were even closer to the periphery (89% CD8 T cells at <30% mean radius length), but here also, their distribution was not modified by any treatment (**Figure 2B**).

### Combined GARP:TGF-β1/PD-1 Blockade Increases Entry of T Cells *via* Intratumoral Vessels in MC38

We then examined whether combined GARP:TGF-β1/PD-1 blockade increased T cell entry *via* intratumoral blood vessels, as reported by Martin et al. for combined latent TGF-β1/PD-1 blockade in MBT-2 tumors (9). Here we used mIF and quantitative imaging of FFPE tumor sections, this time to measure distances between each CD8 T cell and the nearest blood endothelial cell (DAPI^+^CD146^+^ cell, or BEC) and determine the distribution of all CD8 T cells at various distances from their nearest BEC (**Figure 2C**). In murine tissues, CD146 is expressed by blood but not lymphatic endothelial cells (16). In control MC38 tumors, the vast majority of CD8 T cells were <100 µm away from the nearest BEC, with 48% at <30 µm. In mice treated with anti-GARP:TGF-β1, anti-PD-1, or both, the proportions of CD8 T cells at <30 µm from the nearest BEC rose to 55, 61 or 69%, respectively. This suggests that increased infiltration of CD8 T cells in MC38 tumors, after combined GARP:TGF-β1/PD-1 blockade (**Figure 1**), results from increased entry of T cells *via* blood vessels, but that T cells do not migrate further in the tumor bed after extravasation. In control CT26 tumors, 75% of CD8 T cells were at <30 µm from the nearest BEC, and this proportion did not vary with any treatment (**Figure 2C**).

### Combined GARP:TGF-β1/PD-1 Blockade Increases the Density of Blood Vessels Containing GARP^+^ Endothelial Cells Covered by PDGFRβ^+^ Pericytes in MC38, but Not in CT26 Tumors

**Figure 3:**
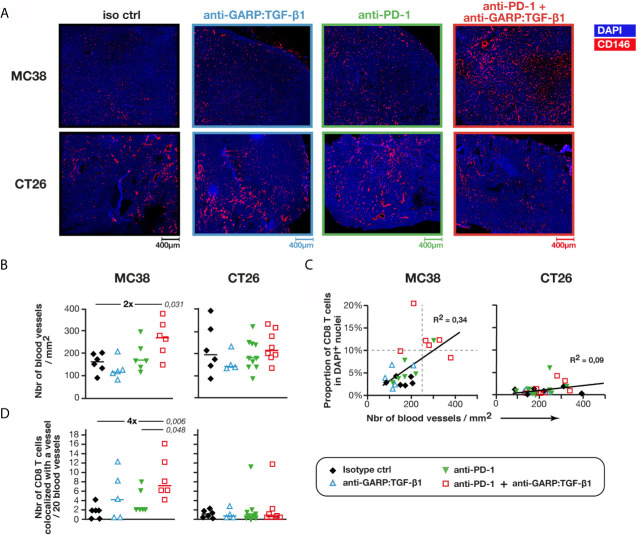
Combined GARP:TGF-β1/PD-1 blockade increases blood vessel density in MC38, but not in CT26 tumors. Sections of FFPE tumors from **Figures 1A, B** analyzed by mIF and quantitative digital imaging. **(A, B)** Representative images and quantification of immunostaining for CD146 (red) and DAPI (blue). Sections were analyzed with the Halo software to identify and quantify the number of blood vessel-like objects (*i.e.* CD146^+^ objects) per mm^2^ of section. Mean of two tumor sections per mouse. **(C)** Correlation between the proportion of CD8 T cells (**Figure 1B**) and the density of blood vessels. Linear regressions and corresponding coefficients of determination (R^2^) are indicated. **(D)** Number of CD8 T cells colocalized with a blood vessel (*i.e.* DAPI^+^CD3^+^CD8^+^CD146^+^ events) per 20 blood vessels. Data points represent the values in individual mice. Horizontal bars: median per group. P values <0.05, calculated with a two-sided Wilcoxon test, are indicated in italics. Numbers in bold: fold-change between the indicated groups. Results from one experiment for MC38 (n = 5–6 mice/group), and pooled from two independent experiments for CT26 (n = 3–6 mice/group).

Next, we examined if increased infiltration of T cells was associated with a higher density of blood vessels in MC38 tumors. Blood vessel-like objects (*i.e.* structures defined based on CD146^+^ surface segmentation) were identified and counted (**Figure 3A**). Median areas of blood vessels were similar in MC38 and CT26 tumors, regardless of treatment (**Figure S4**). Densities of blood vessel-like objects in control MC38 and CT26 tumors were also similar, with 161 ± 54 (median ± IQR) blood vessels per mm^2^ in MC38, and 196 ± 132 in CT26 tumors (**Figure 3B**). Unexpectedly, we observed that combined GARP:TGF-β1/PD-1 blockade increased blood vessel density about 2-fold in MC38 (272 ± 91), but not in CT26 tumors (**Figure 3B**). Blood vessel densities correlated with proportions of tumor-infiltrating CD8 T cells in MC38, but not in CT26 tumors. Three of the four MC38 tumors with the highest CD8 T cell infiltration (>10% CD8 T cells in DAPI^+^ cells) and the highest blood vessel density (>250 vessels/mm^2^) were from mice treated with anti-GARP:TGF-β1 + anti-PD-1 (**Figure 3C**). We counted a median of about 1.8 and 1.2 CD8 T cell in every 20 vessels of untreated MC38 and CT26 tumors, respectively, and this number increased 4-fold after combined GARP:TGF-β1/PD-1 blockade in MC38, but not in CT26 tumors (**Figure 3D**). Thus, increased T cell infiltration in MC38 tumors upon combined GARP:TGF-β1/PD-1 blockade results from both increased blood vessel density and increased CD8 T cell extravasation into the tumors.

**Figure 4:**
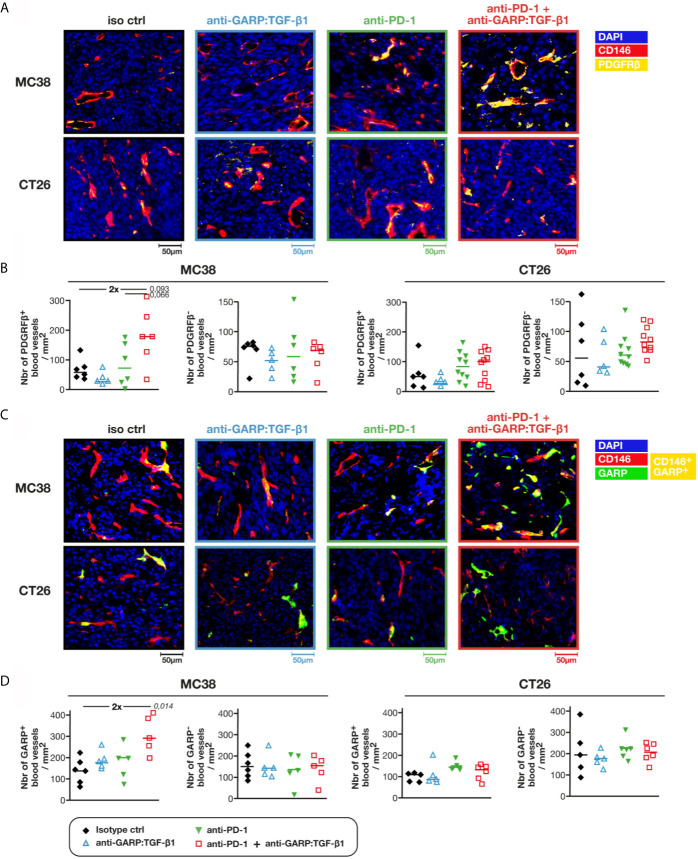
Combined GARP:TGF-β1/PD-1 blockade increases the density of intratumoral GARP^+^ blood vessels covered by pericytes in MC38, but not in CT26 tumors. Additional FFPE and frozen tumor sections obtained from mice in **Figures 1A, B** were analyzed by mIF and quantitative digital imaging. **(A, B)** Representative images **(A)** and quantification **(B)** of PDGFRβ^+^ and PDGFRβ^-^ blood vessels (*i.e.* CD146^+^PDGFRβ^+^ and CD146^+^PDGFRβ^−^ objects) in FFPE tumor sections stained with anti-CD146 (red), anti-PDGFRβ (yellow) and Hoescht (DAPI, blue). **(C, D)** Representative images **(C)** and quantification **(D)** of GARP^+^ and GARP^-^ blood vessels (*i.e.* CD146^+^GARP^+^ and CD146^+^GARP^−^ objects) per mm^2^ of frozen tumor sections stained with anti-CD146 (red), anti-GARP (green) and Hoescht (DAPI, blue). GARP^+^ vessels appear in yellow. Data points represent values in individual mice. Horizontal bars: median per group. P values <0.05 are indicated in italics (calculated with a two-sided Wilcoxon test). Numbers in bold: fold-change between the indicated groups. One tumor section analyzed per mouse.

Intratumoral blood vessels are often described as structurally abnormal, displaying loose cellular junctions, high number of fenestrations and discontinuous basement membrane, which can impair lymphocyte extravasation (17). To measure the functionality and stability of blood vessels, we used anti-PDGFRβ antibodies to identify blood vessels covered by pericytes (*i.e.* CD146^+^PDGFRβ^+^ objects) (**Figure 4A**). We observed a 2-fold increase in the density of PDGFRβ^+^ blood vessels in MC38, but not in CT26 tumors, upon GARP:TGF-β1/PD-1 blockade as compared with controls or anti-PD-1 alone (**Figure 4B**). No increase in the density of PDGFRβ-negative blood vessels was observed in either MC38 or CT26 tumors, whatever the treatment (**Figure 4B**). Thus, increased blood vessel density in MC38 tumors after GARP:TGF-β1/PD-1 blockade can be attributed to an increased density of those vessels that are stabilized by pericytes, a parameter often taken as an indicator of blood vessel normalization in tumors (17).

GARP:TGF-β1 complexes are known to be expressed on endothelial cells (2, 8, 18). We therefore verified whether treatment with anti-GARP:TGF-β1 mAbs modified the densities and proportions of blood vessels containing GARP^+^ BECs in frozen MC38 and CT26 tumors (**Figure 4C**), as GARP can be detected by mIF in frozen tissues only (8). In untreated tumors, 45.3% ± 6.2% (median ± IQR) of blood vessels contained GARP^+^ BECs in MC38, versus 30.7% ± 7.7% in CT26 tumors. Combined GARP:TGF-β1/PD-1 blockade increased GARP^+^ blood vessel density about 2-fold in MC38, but not in CT26 tumors (**Figure 4D**). Densities of GARP-negative blood vessels did not change in MC38 or CT26 tumors, whatever the treatment. In a follow up experiment, we co-stained frozen tumor sections for GARP and PDGFRβ. The two markers were frequently co-expressed in intratumoral blood vessels: whilst 63.8% ± 5.8% (median ± IQR) of GARP^+^ blood vessels were also PDGFRβ^+^ in MC38 tumors, only 13.6% ± 4.2% of GARP^-^ vessels expressed PDGFRβ (**Figure S5**). We conclude that combined GARP:TGF-β1/PD-1 blockade increased the density of GARP^+^ blood vessels covered by pericytes in MC38, but not in CT26 tumors.

### Active TGF-β1 Inhibits the Proliferation of Murine GARP^+^ Endothelial Cells *In Vitro*

**Figure 5:**
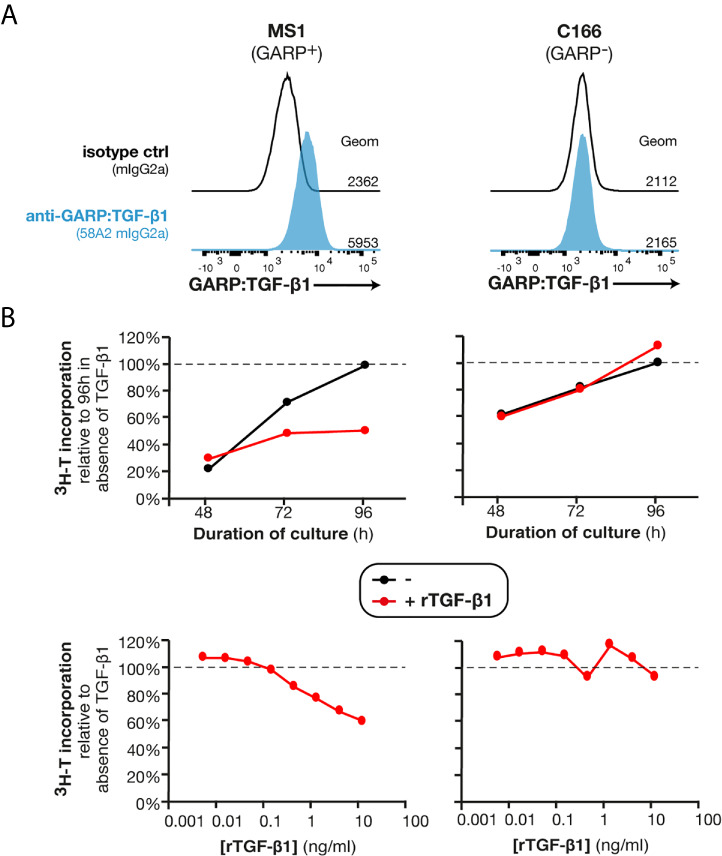
Recombinant TGF-β1 exerts a cytostatic effect on GARP^+^ endothelial cells in *vitro*. **(A)** Flow cytometry analyses of MS1 and C166 murine endothelial cell lines stained with anti-GARP:TGF-β1 antibody (clone 58A2) or isotype control. Histograms are gated on live single cells. Geom, geometric mean fluorescence intensity. **(B)** C166 and MS1 cells were seeded in presence or absence of rTGF-β1 (top: 1 ng/ml; bottom: indicated concentrations) and cultivated during 48, 72 or 96 h (top) or 96 h (bottom). Tritiated thymidine (^3^H-T) was added during the last 24 h of culture, and cells were collected to measure ^3^H-T incorporation as a read-out of proliferation. Data points represent means of triplicate wells. Results are representative of three independent experiments.

TGF-β1 exerts a cytostatic effect on many cell types. By blocking TGF-β1 activation on the surface of GARP^+^ endothelial cells, anti-GARP:TGF-β1 mAbs could increase endothelial cell proliferation and the formation of new GARP^+^PDGFRβ^+^ functional blood vessels in MC38 tumors. *In vitro* proliferation of MS1, an endothelial cell line which is derived from a pancreatic sarcoma and expressing surface GARP:TGF-β1 complexes (**Figure 5A**), was inhibited by recombinant active TGF-β1 (rTGF-β1) in a dose-dependent manner (**Figure 5B**). Noteworthy, incubation with neutralizing anti-active TGF-β1,2,3 (clone 1D11) or blocking anti-GARP:TGF-β1 (clone 58A2) mAbs did not increase MS1 proliferation (**Figure S6**), indicating that they are not under the influence of autocrine TGF-β1 activity. This result indicates that in the absence of a stimulus or another cell type, MS1 endothelial cells are unable to activate the latent TGF-β1 presented by GARP on their surface. We also tested the effect of rTGF-β1 on another murine endothelial cell line, C166, which originates from yolk sac. We observed no inhibition of proliferation (**Figures 5A, B**).

The inhibition of MS1 proliferation by rTGF-β1 *in vitro* suggests that anti-GARP:TGF-β1 mAbs could favor the proliferation of GARP^+^ endothelial cells *in vivo*, and consequently increase blood vessel densities in MC38 tumors.

### Combined GARP:TGF-β1/PD-1 Blockade Increases Expression of E-Selectin by BECs in MC38, but Not in CT26 Tumors

**Figure 6:**
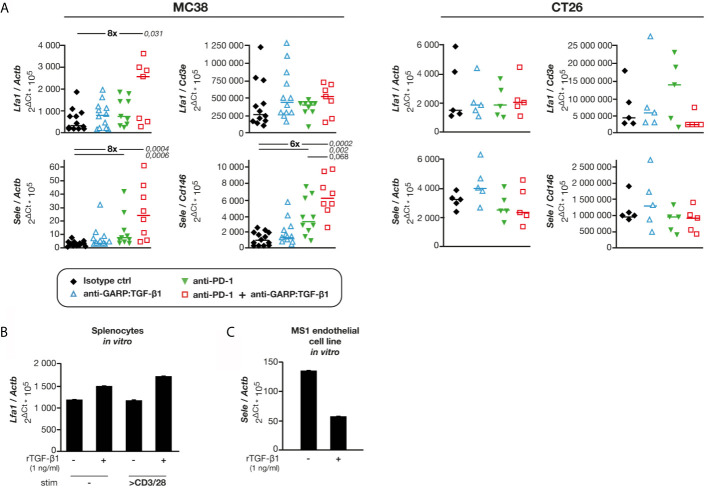
Combined GARP:TGF-β1/PD-1 blockade increases expression of *Sele* and *Lfa1* in MC38, but not in CT26 tumors. Tumor samples shown in **Figure 1C** were analyzed by RT-qPCR. **(A)** mRNA levels of *Lfa1* and *Sele* relative to *Actb* (housekeeping gene), *Cd3e* (T-cell specific gene) or *Cd146* (endothelial cell-specific gene). Data points represent values in one mouse. Horizontal bars: median per group. P values <0.05, as calculated with a two-sided Wilcoxon test, are indicated in italics. Numbers in bold: fold-change between the indicated groups. **(B)** Expression of *Lfa1* in BALB/c splenocytes exposed to rTGF-β, in presence or absence of anti-CD3/CD28 beads during 24 h *in vitro*. Bars represent means (+SD) of duplicates. Results from one experiment. **(C)** Expression of *Sele* in C166 and MS1 cells exposed to rTGF-β during 24 h *in vitro*. Bars represent means (+SD) of duplicates. Results from one experiment.

TGF-β1 regulates expression of adhesion molecules in hematopoietic and endothelial cells (19–21). We measured the expression of genes encoding various adhesion molecules in MC38 and CT26 tumor samples from control and mAb-treated mice. No difference was observed for expression of *Vcam1* and *Icam1* relative to *Actb* in any treatment group as compared with controls (**Figure S7**). In contrast, expression of *Lfa1* and *Sell*, encoding adhesion molecules expressed on leukocytes, and that of *Sele*, encoding E-selectin expressed on BECs, were increased after combined GARP:TGF-β1/PD-1 blockade in MC38, but not in CT26 tumors (**Figure 6A** and **Figure S7**). We measured *Lfa1/Cd3e, Sell/Cd3e* and *Sele/Cd146* mRNA ratios, to normalize adhesion molecule expression to the abundance of CD3 T cells or CD146^+^ BECs (**Figure 6A** and **Figure S7**). No significant difference in *Lfa1/Cd3e* or *Sell/Cd3e* ratios was observed in MC38 samples from control or mAb-treated mice. Thus, increased expression of these genes relative to *Actb* after GARP:TGF-β1/PD-1 blockade in MC38 tumors is probably mostly due to increased T cell infiltration. This was different for expression of *Sele*: ratios of *Sele/Actb* and *Sele/Cd146* were significantly increased after combined GARP:TGF-β1/PD-1 blockade in MC38, but not in CT26 tumors. This suggests that increased *Sele* expression in MC38 tumors results not only from increased BEC densities but also from increased *Sele* mRNA levels per BEC. We verified whether exposure to rTGF-β1 regulates *Lfa1* or *Sele* expression by murine activated T cells or endothelial cells *in vitro*, respectively. rTGF-β1 marginally increased (1.3- to 1.5-fold) *Lfa1* expression by murine splenocytes stimulated or not with anti-CD3/CD28 beads (**Figure 6B**). In contrast, rTGF-β1 reduced *Sele* expression by more than 2-fold in MS1 endothelial cells (**Figure 6C**), confirming previous observations on HUVECs and murine lung, heart and liver endothelial cells (22, 23).

## Discussion

Altogether, our results suggest that combined GARP:TGF-β1/PD-1 blockade exerts anti-tumor activity in MC38 tumors by increasing the density of intratumoral GARP^+^ blood vessels covered by PDGFRβ^+^ pericytes, the expression of E-selectin by BECs, and the extravasation and infiltration of T cells, including activated anti-tumor CD8 T cells.

Because rTGF-β1 inhibited the proliferation of GARP^+^ endothelial cells *in vitro*, we propose that anti-GARP:TGF-β1 mAbs act by inhibiting TGF-β1-mediated endothelial cell cytostasis in MC38 tumors, thereby favoring BEC proliferation and densification of the intratumoral blood vasculature. Anti-PD-1 mAbs, alone or in combination with anti-CTLA-4, were previously shown to increase pericyte coverage of blood vessels in mouse tumors, but these treatments did not increase, and even reduced intratumoral blood vessel densities (24–27). Combining anti-PD-1 with anti-GARP:TGF-β1 could therefore increase pericyte coverage of blood vessels as a result of PD-1 blockade, while also increasing BEC proliferation and blood vessel density as a result of GARP:TGF-β1 blockade. This would explain the densification of pericyte-covered blood vessels observed in MC38 tumors upon combined GARP:TGF-β1/PD-1 blockade. Increased density of pericyte-covered blood vessels is an indicator of tumor vasculature normalization, which could improve tumor perfusion (17, 28). Other approaches targeting the tumor vasculature to overcome resistance to PD-1/PD-L1 blockade were tested previously, *e.g.* with anti-VEGFR2 mAbs, which enhanced vascular normalization but did not increase blood vessel density in mouse models of breast and pancreatic cancer (29). Anti-GARP:TGF-β1 could represent a more efficient and safer alternative, densifying in addition to normalizing the blood vasculature by targeting GARP-expressing cells only.

The cellular source of the active TGF-β1 exerting cytostatic effects on BECs in MC38 tumors is not yet clearly identified. We observed that approximately half of tumor blood vessels in MC38 tumors contained endothelial cells expressing GARP:TGF-β1 on their surface. It would thus be tempting to speculate that anti-GARP:TGF-β1 improves the anti-tumor efficacy of anti-PD-1 by blocking TGF-β1 activation on the surface of GARP^+^ endothelial cells. However, we observed that *in vitro*, endothelial cells were not able to activate latent TGF-β1 presented by GARP on their surface. This suggests that an unidentified stimulus, or another cell type, may be required to allow TGF-β1 activation on the surface of endothelial cells *in vivo*. In T cells, the mere presence of surface GARP:TGF-β1 is not sufficient to induce TGF-β1 activation. TGF-β1 activation by Tregs requires TCR stimulation and interaction of GARP:TGF-β1 complexes with integrin αVβ8 (3). The situation might be similar for endothelial cells, which would require an additional stimulus and/or the intervention of an αVβ8-expressing cell type to activate latent TGF-β1 presented by GARP on their surface *in vivo*. Tregs themselves, entering the tumor *via* blood vessels, represent interesting candidates. Indeed, we previously showed that the anti-tumor activity of combined GARP:TGF-β1/PD-1 blockade was lost in Treg-specific *Garp* knock-out mice bearing MC38 tumors, suggesting that targeting GARP-expressing Tregs is indispensable for anti-GARP:TGF-β1 to exert anti-tumor activity (8). It could thus very well be that the cellular source of the active TGF-β1 exerting cytostasis on BECs in MC38 tumors is the immunosuppressive Tregs. Considering that tumor-infiltrating T cells, which include Tregs, are more abundant in MC38 than in CT26 tumors, this could explain at least in part why GARP:TGF-β1 blockade increases the density of the tumor blood vasculature in MC38, but not in CT26 tumors.

Whichever the cellular source of TGF-β1 signals blocked by anti-GARP:TGF-β1, increased tumor perfusion will increase T cell influx in the MC38 tumor vasculature. We observed that expression of *Sele*, a gene encoding E-selectin and repressed by TGF-β1, was increased in MC38 tumors upon combined GARP:TGF-β1/PD-1 blockade. E-selectin on endothelial cells allows for the adhesion of activated leukocytes and facilitates their extravasation by slowing down their rolling on blood vessel walls. Therefore, anti-GARP:TGF-β1 mAbs, by blocking TGF-β1 activity and increasing *Sele* expression, could facilitate T cell extravasation and infiltration within MC38 tumors.

It is noteworthy that activated intratumoral T cells can themselves enhance vascular functionality by secreting TNFα, known to upregulate E-selectin on blood endothelial cells (23), and IFNγ, which induces expression of chemokines involved in pericyte recruitment (25, 30, 31). We observed a significant increase in *Ifng* mRNA levels and proportions of IFNγ-producing CD4 T cells in MC38 tumors after combined GARP:TGF-β1/PD-1 blockade. Thus, increased tumor blood vessel density and functionality induced by combined GARP:TGF-β1/PD-1 blockade could be further reinforced as a consequence of increased infiltration by activated T cells, in a positive feedback loop.

The mode of action of combined GARP:TGF-β1/PD-1 blockade in MC38 tumors contrasts with that previously described in CT26 tumors, in which it increased effector functions of already present anti-tumor CD8 T cells, without increasing T cell infiltration or blood vessel density. It is unclear why combined GARP:TGF-β1/PD-1 blockade did not densify the blood vasculature and increase T cell infiltration in CT26 tumors, and conversely, why it did not increase effector functions of tumor-infiltrating CD8 T cells in MC38 tumors. Whether different modes of action result from using different colon carcinoma cell lines (MC38 *vs* CT26) or different syngeneic mouse strains (C57BL/6 *vs* BALB/c, respectively) could be assessed by comparing the two tumor models in F1 mice. The proportion of blood vessels containing GARP^+^ BECs in CT26 tumors was lower as compared with MC38 tumors, perhaps contributing to the lack of effect of anti-GARP:TGF-β1 on the blood vasculature in these tumors. Nevertheless, combined GARP:TGF-β1/PD-1 blockade increased immune-mediated tumor rejections to a similar extent in CT26 and MC38 tumors (8), indicating that both modes of action can significantly contribute to the anti-tumor activity of the combination.

Taken together, our results suggest that combined GARP:TGF-β1/PD-1 blockade can exert anti-tumor activity *via* multiple mechanisms, not only by increasing effector functions of anti-tumor T cells already present within tumors, but also by increasing tumor blood vessel density and infiltration by new anti-tumor T cells. Anti-GARP:TGF-β1 mAbs could thus be tested in the clinics to overcome resistance to PD-1/PD-L1 blockade in patients with a broad range of cancer types, including not only tumors already infiltrated by T cells, but also poorly vascularized tumors with low immune cell infiltration.

## Data Availability Statement

The original contributions presented in the study are included in the article/**Supplementary Material**. Further inquiries can be directed to the corresponding author.

## Ethics Statement

The animal study was reviewed and approved by Comité d’Ethique pour l’Expérimentation Animale Secteur des Sciences de la Santé Université catholique de Louvain.

## Author Contributions

SL conceived the study. CB, PM, GS, AN, PC, NB, and SL analyzed the data. CB, PM, GS, NV-B, FB, and LR performed experiments. SL and CB wrote the manuscript. All authors contributed to the article and approved the submitted version.

## Funding

This work was supported by grants from the Fondation contre le Cancer (grant F/2016/837), from the European Research Council (ERC) under the European Union’s Horizon 2020 research and innovation program (grant TARG-SUP 682818), from the Actions de Recherche Concertées (grant 14/19-056), from the Fonds National de la Recherche Scientifique (PDR number T.0089.16), from the F.N.R.S.-Télévie (PDR-TLV number 7.8511.19) and from Région Wallonne (program WALinnov, project IMMUCAN, convention number 1610119). This work was also supported by grants from Walloon Excellence in Life Sciences and Biotechnology (WELBIO), Wavre, Belgium (CR-2019A-02). CB was supported by a F.N.R.S.-Télévie grant and a fellowship from the UCLouvain. PM and LR were supported by a F.N.R.S.-Télévie grant, and GS was supported by a FRIA fellowship (F.R.S.-F.N.R.S.).

## Conflict of Interest

Patents pertaining to results obtained with the 58A2 antibody clone (anti-mouse GARP:TGF-β1) have been filed under the Patent Cooperation Treaty (International application Number PCT/IB2019/053753), with SL, GS, PC as inventors and UCLouvain and argenx as applicants. SL received research support from argenx and owns stock options in argenx.

The remaining authors declare that the research was conducted in the absence of any commercial or financial relationships that could be construed as a potential conflict of interest.

## Publisher’s Note

All claims expressed in this article are solely those of the authors and do not necessarily represent those of their affiliated organizations, or those of the publisher, the editors and the reviewers. Any product that may be evaluated in this article, or claim that may be made by its manufacturer, is not guaranteed or endorsed by the publisher.

## Supplementary Material

Supplementary Figure 1 Combined GARP:TGF-β1/PD-1 blockade and anti-PD-1 alone reduce growth of MC38 and CT26 tumors. Volume and weight of tumors collected on day 13 for analyses shown in **Figures 1** **–** **4** and **Figure 6**. Data points represent values in individual mice. Horizontal bars: median per group. P values < 0.05, as calculated with a two-sided Wilcoxon test, are indicated by numbers in italics. Results from one experiment for MC38 (n=5-6 mice/group), and pooled from two independent experiments for CT26 (n=3-6 mice/group in each experiment).

Supplementary Figure 2 CD8 T cells infiltrating MC38 tumors include activated anti-tumor T cells, but combined GARP:TGF-β1/PD-1 blockade does not increase their proportions or functions. MC38 tumors from mice treated as indicated in **Figure 1** were collected on day 13 to perform flow cytometry and RT-qPCR. **(A)** Proportions (%) of the indicated cell types among tumor infiltrating leukocytes (CD45^+^) or CD8 T cells (CD45^+^ CD8^+^). Results from one experiment (n=6-10 mice/group). **(B)** Proportions (%) of cells producing IFNγ, TNFα, and/or expressing surface CD107a among the indicated tumor-infiltrating cell subsets, obtained on unstimulated bulk processed tumors. Results from one experiment (n=6-10 mice/group). **(C)** Expression of *Ifng*, *Tnfa*, *Prf1* and *Gzmb* relative to *Actb* in MC38 tumors. Results from 2 independent experiments (n=4-7 mice/group in each experiment). Data points represent values in individual mice. Horizontal bars: median per group. P values < 0.05, as calculated with a two-sided Wilcoxon test are indicated in italics. Numbers in bold: fold-change between the indicated groups.

Supplementary Figure 3 Combined GARP:TGF-β1/PD-1 blockade does not increase the proliferation of tumor infiltrating CD8 T cells in MC38 and CT26. MC38 tumors from mice treated as indicated in **Figure 1** were collected on day 13. CT26 tumors from mice treated as indicated in **Figure 1** (with the exception that antibodies were injected on days 6, 10 and 14) were collected on day 14. Tumors were dissociated and analyzed by flow cytometry. Results from one experiment for MC38 (n=5 mice/group) and one experiment for CT26 (n=4/group). **(A)** Percentage of Ki-67^+^ cells in live single CD8 T cells (CD45^+^CD8^+^ for MC38, and CD3^+^CD8^+^ for CT26). **(B)** Geometric mean fluorescence intensity (geomean) for Ki-67 staining in live single Ki-67^+^ CD8 T cells. Data points represent values in one mouse. Horizontal bars: median per group.

Supplementary Figure 4 Combined GARP:TGF-β1/PD-1 blockade does not modify mean area of blood vessels in MC38 and CT26 tumors. mIF and quantitative digital imaging of sections shown in **Figure 4**. Mean area of CD146^+^ objects in tumor sections (n=134 to 26 939 CD146^+^ objects per section). Data points represent the mean values of two tumor sections analyzed in individual mice. Horizontal bars: median per group.

Supplementary Figure 5 A majority of GARP^+^ blood vessels also express PDGFRβ in MC38 tumors. Frozen MC38 tumor sections from mice shown in **Figures 1A, B** were used for mIF and quantitative imaging. **(A)** Representative image of a MC38 control tumor stained with anti-CD146 antibody (red), anti-PDGFRβ (yellow) and anti-GARP (green). Left: CD146 and PDGFRβ signals. Right: GARP and PDGFRβ signals on the same area. White arrow: CD146^+^PDGFRβ^+^GARP^+^ vessel (top images) or CD146^+^PDGFRβ^+^GARP^-^ vessel (bottom images). **(B)** Proportion (%) of PDGFRβ^+^ vessels in GARP^-^ or GARP^+^ blood vessels. Data points represent values for individual mice. Horizontal bars: median per group. P value < 0.05, as calculated with a two-sided Wilcoxon test, are indicated in italics. Number in bold: fold-change between the indicated groups. One tumor section analyzed per mouse.

Supplementary Figure 6 C166 and MS1 endothelial cells do not activate TGF-β1 in *vitro* in absence of stimulus or another cell type. C166 and MS1 cells were used to perform a proliferation assay as described in **Figure 5B**. Incorporation of ^3^H-T was measured after 96 hours of culture in the presence of blocking anti-GARP:TGF-β1 (clone 58A2) or neutralizing anti-TGF-β1, β2, β3 (clone 1D11) mAbs. Bars indicate means (+ SD) for triplicate wells. Data representative of 3 independent experiments.

Supplementary Figure 7 Combined GARP:TGF-β1/PD-1 blockade increases expression of *Sell* but not of *Icam1* and *Vcam1* in MC38 tumors. RT-qPCR analyses relative to **Figure 6A**. Graphs show the expression level of *Icam1*, *Vcam1* and *Sell* normalized by *Actb*, *Cd3e* or *Cd146*. Data points represent values measured in individual mice. Horizontal bars: median per group. Number in italics indicate P values < 0.05 as calculated with a two-sided Wilcoxon test, and numbers in bold indicate fold-changes between groups for selected comparisons.

Supplementary Table 1 Primers and probes sequences.
