# Supplementary figures and images for: Combined Blockade of GARP:TGF-β1 and PD-1 Increases Infiltration of T Cells and Density of Pericyte-Covered GARP+ Blood Vessels in Mouse MC38 Tumors
Source: Front Immunol. 2021 Jul 27;12:704050. doi: 10.3389/fimmu.2021.704050 (PMC8353334; doi:10.3389/fimmu.2021.704050)

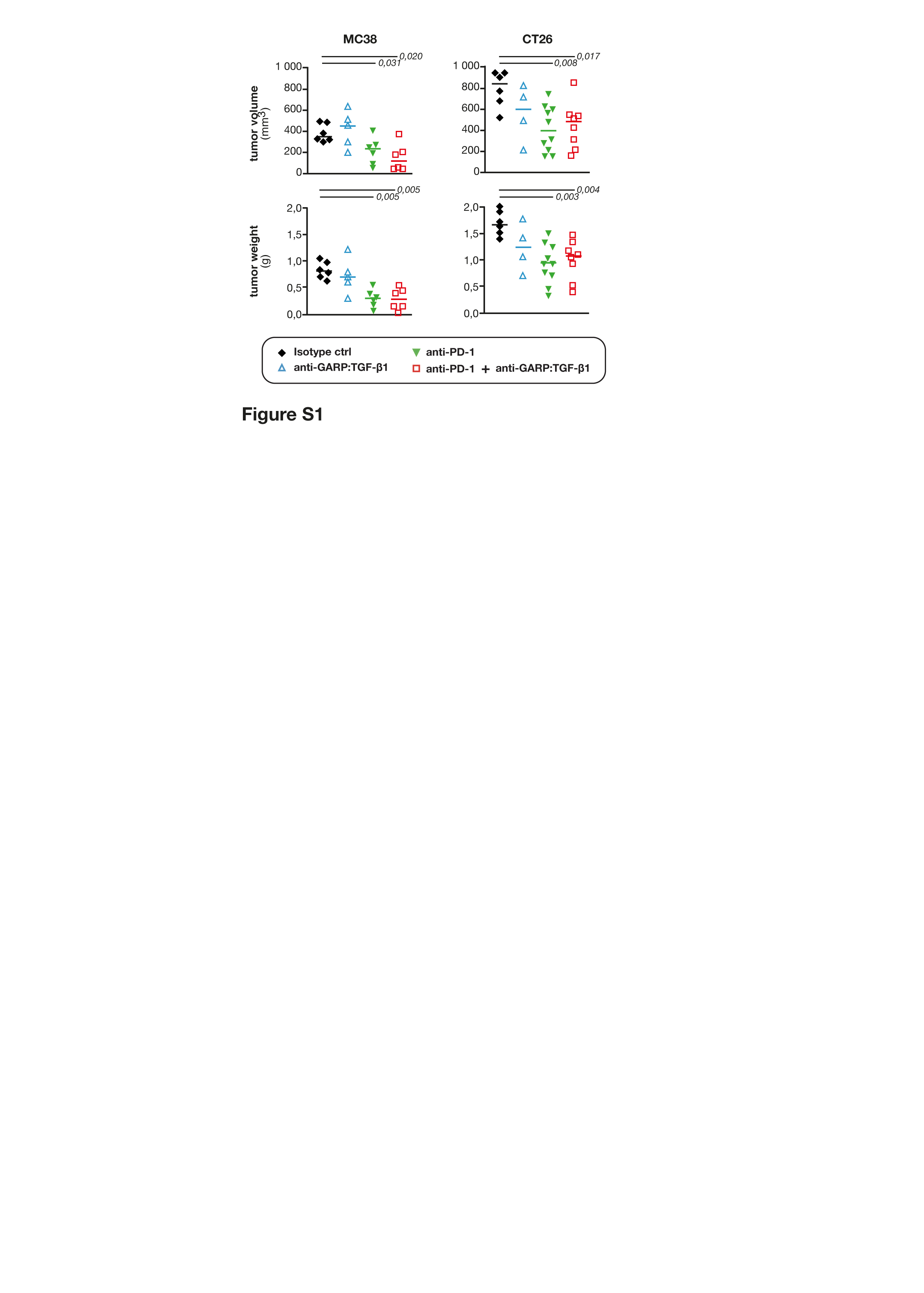

Supplement: Supplementary Figure 1 — Combined GARP:TGF-β1/PD-1 blockade and anti-PD-1 alone reduce growth of MC38 and CT26 tumors. Volume and weight of tumors collected on day 13 for analyses shown in Figures 1 – 4 and Figure 6. Data points represent values in individual mice. Horizontal bars: median per group. P values < 0.05, as calculated with a two-sided Wilcoxon test, are indicated by numbers in italics. Results from one experiment for MC38 (n=5-6 mice/group), and pooled from two independent experiments for CT26 (n=3-6 mice/group in each experiment). [file Image_1.tif]

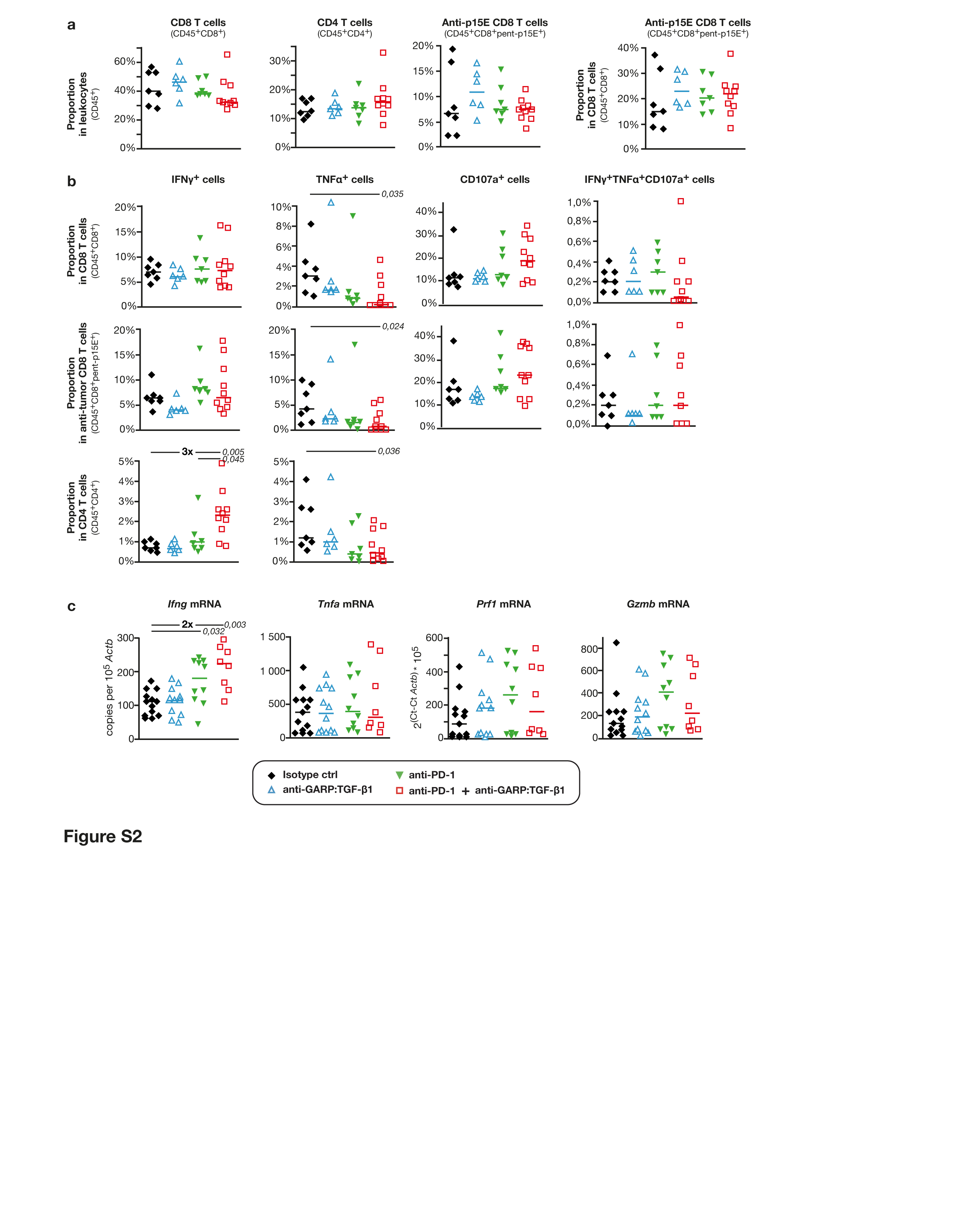

Supplement: Supplementary Figure 2 — CD8 T cells infiltrating MC38 tumors include activated anti-tumor T cells, but combined GARP:TGF-β1/PD-1 blockade does not increase their proportions or functions. MC38 tumors from mice treated as indicated in Figure 1 were collected on day 13 to perform flow cytometry and RT-qPCR. (A) Proportions (%) of the indicated cell types among tumor infiltrating leukocytes (CD45+) or CD8 T cells (CD45+ CD8+). Results from one experiment (n=6-10 mice/group). (B) Proportions (%) of cells producing IFNγ, TNFα, and/or expressing surface CD107a among the indicated tumor-infiltrating cell subsets, obtained on unstimulated bulk processed tumors. Results from one experiment (n=6-10 mice/group). (C) Expression of Ifng, Tnfa, Prf1 and Gzmb relative to Actb in MC38 tumors. Results from 2 independent experiments (n=4-7 mice/group in each experiment). Data points represent values in individual mice. Horizontal bars: median per group. P values < 0.05, as calculated with a two-sided Wilcoxon test are indicated in italics. Numbers in bold: fold-change between the indicated groups. [file Image_2.tif]

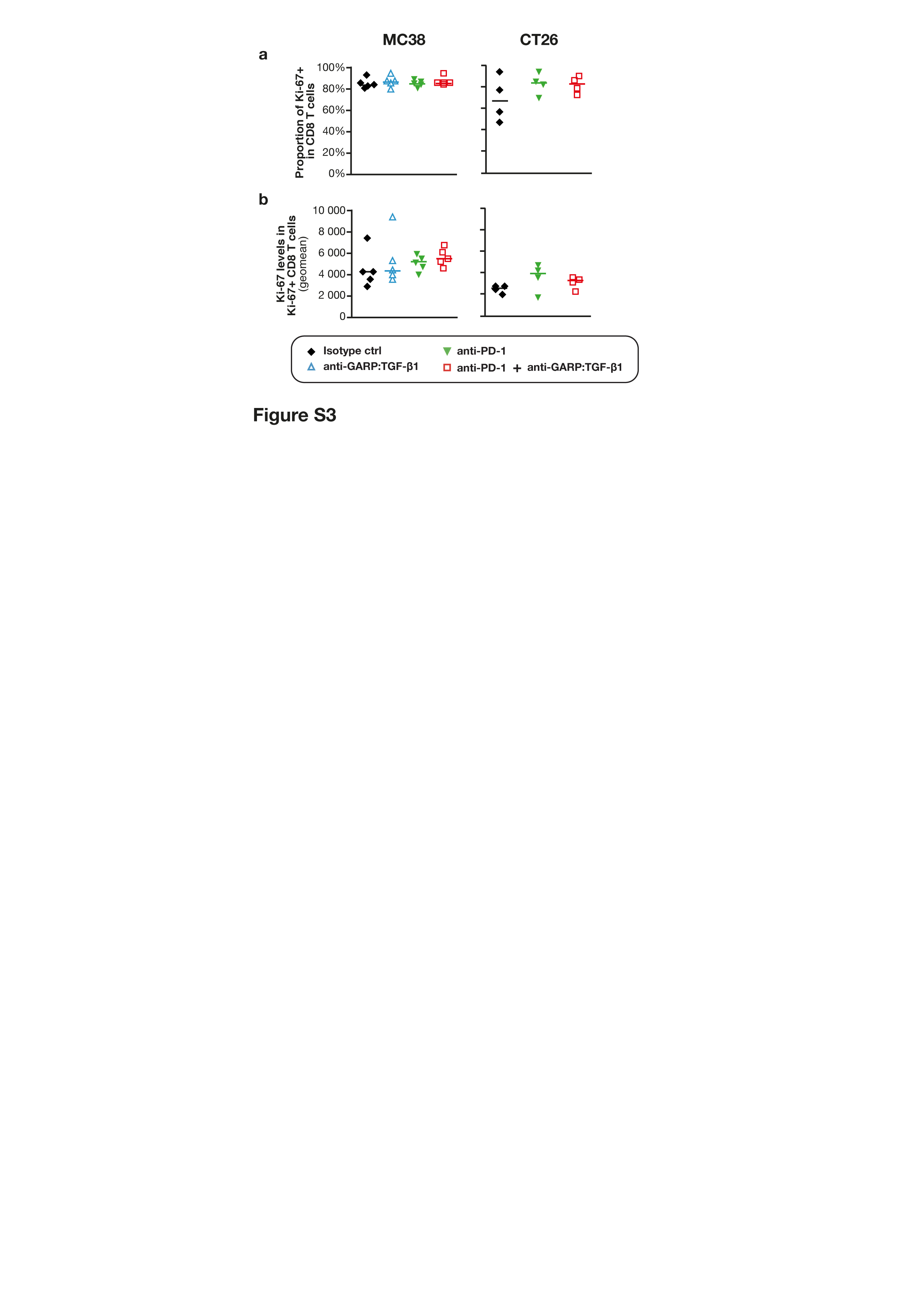

Supplement: Supplementary Figure 3 — Combined GARP:TGF-β1/PD-1 blockade does not increase the proliferation of tumor infiltrating CD8 T cells in MC38 and CT26. MC38 tumors from mice treated as indicated in Figure 1 were collected on day 13. CT26 tumors from mice treated as indicated in Figure 1 (with the exception that antibodies were injected on days 6, 10 and 14) were collected on day 14. Tumors were dissociated and analyzed by flow cytometry. Results from one experiment for MC38 (n=5 mice/group) and one experiment for CT26 (n=4/group). (A) Percentage of Ki-67+ cells in live single CD8 T cells (CD45+CD8+ for MC38, and CD3+CD8+ for CT26). (B) Geometric mean fluorescence intensity (geomean) for Ki-67 staining in live single Ki-67+ CD8 T cells. Data points represent values in one mouse. Horizontal bars: median per group. [file Image_3.tif]

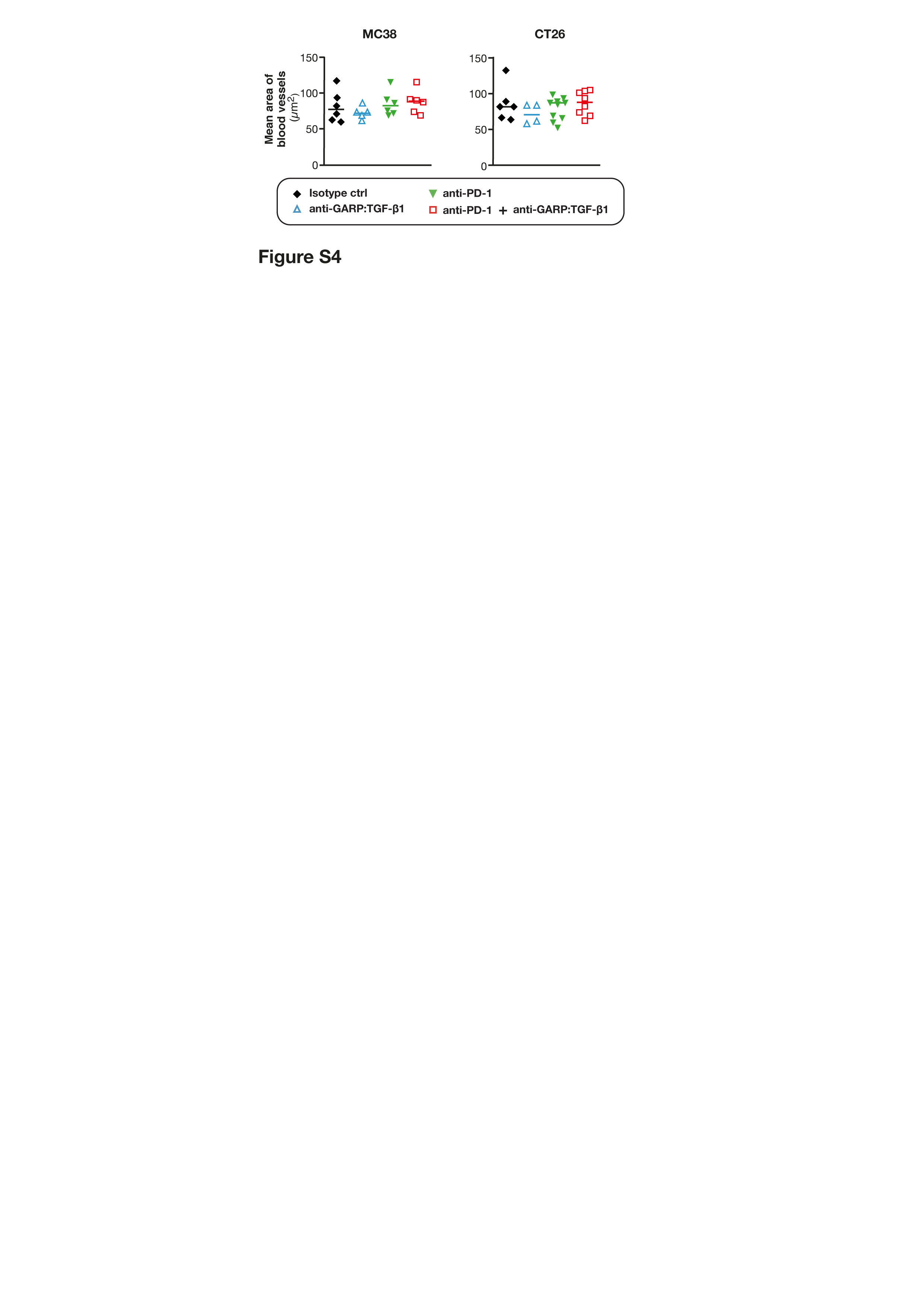

Supplement: Supplementary Figure 4 — Combined GARP:TGF-β1/PD-1 blockade does not modify mean area of blood vessels in MC38 and CT26 tumors. mIF and quantitative digital imaging of sections shown in Figure 4. Mean area of CD146+ objects in tumor sections (n=134 to 26 939 CD146+ objects per section). Data points represent the mean values of two tumor sections analyzed in individual mice. Horizontal bars: median per group. [file Image_4.tif]

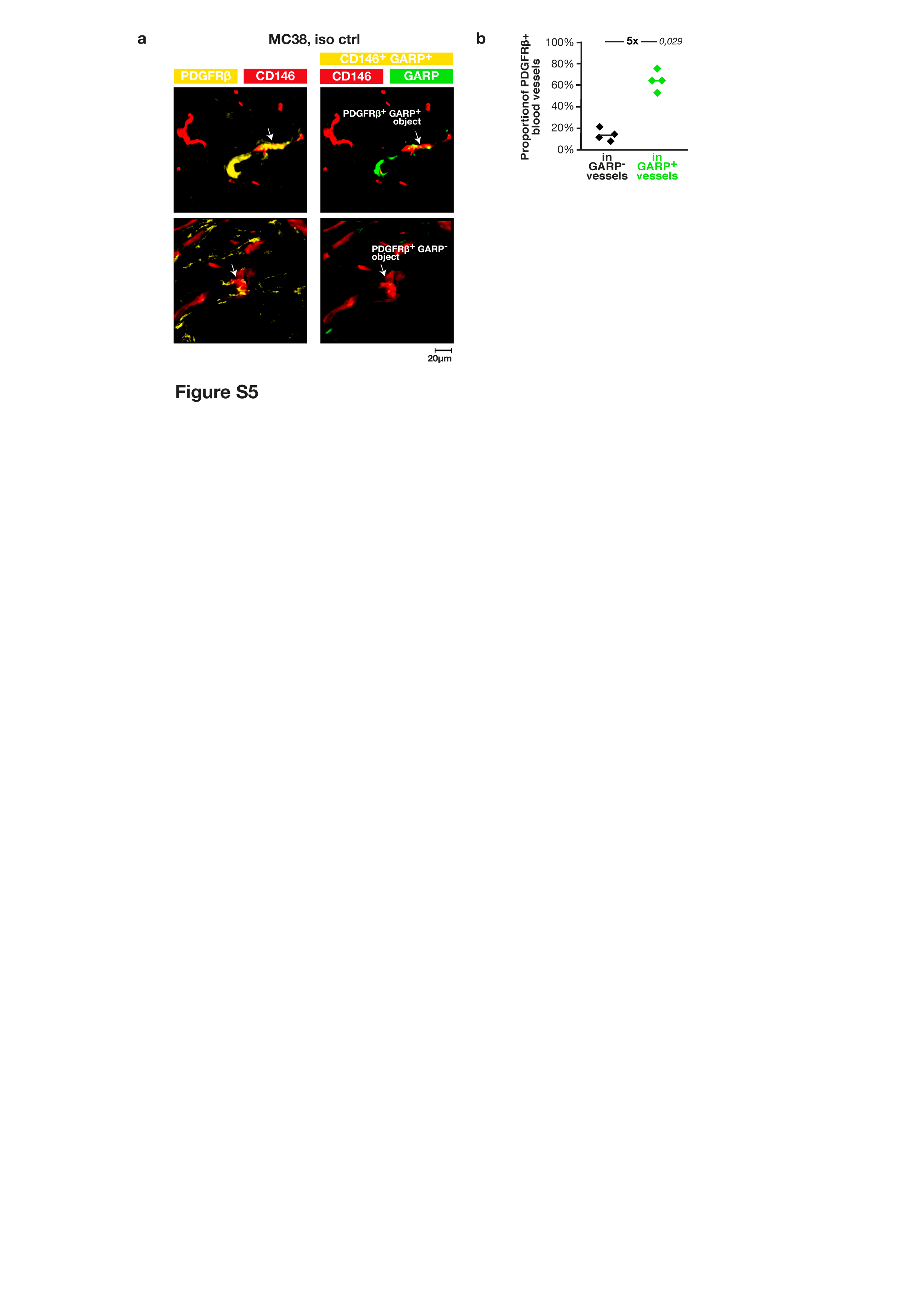

Supplement: Supplementary Figure 5 — A majority of GARP+ blood vessels also express PDGFRβ in MC38 tumors. Frozen MC38 tumor sections from mice shown in Figures 1A, B were used for mIF and quantitative imaging. (A) Representative image of a MC38 control tumor stained with anti-CD146 antibody (red), anti-PDGFRβ (yellow) and anti-GARP (green). Left: CD146 and PDGFRβ signals. Right: GARP and PDGFRβ signals on the same area. White arrow: CD146+PDGFRβ+GARP+ vessel (top images) or CD146+PDGFRβ+GARP- vessel (bottom images). (B) Proportion (%) of PDGFRβ+ vessels in GARP- or GARP+ blood vessels. Data points represent values for individual mice. Horizontal bars: median per group. P value < 0.05, as calculated with a two-sided Wilcoxon test, are indicated in italics. Number in bold: fold-change between the indicated groups. One tumor section analyzed per mouse. [file Image_5.tif]

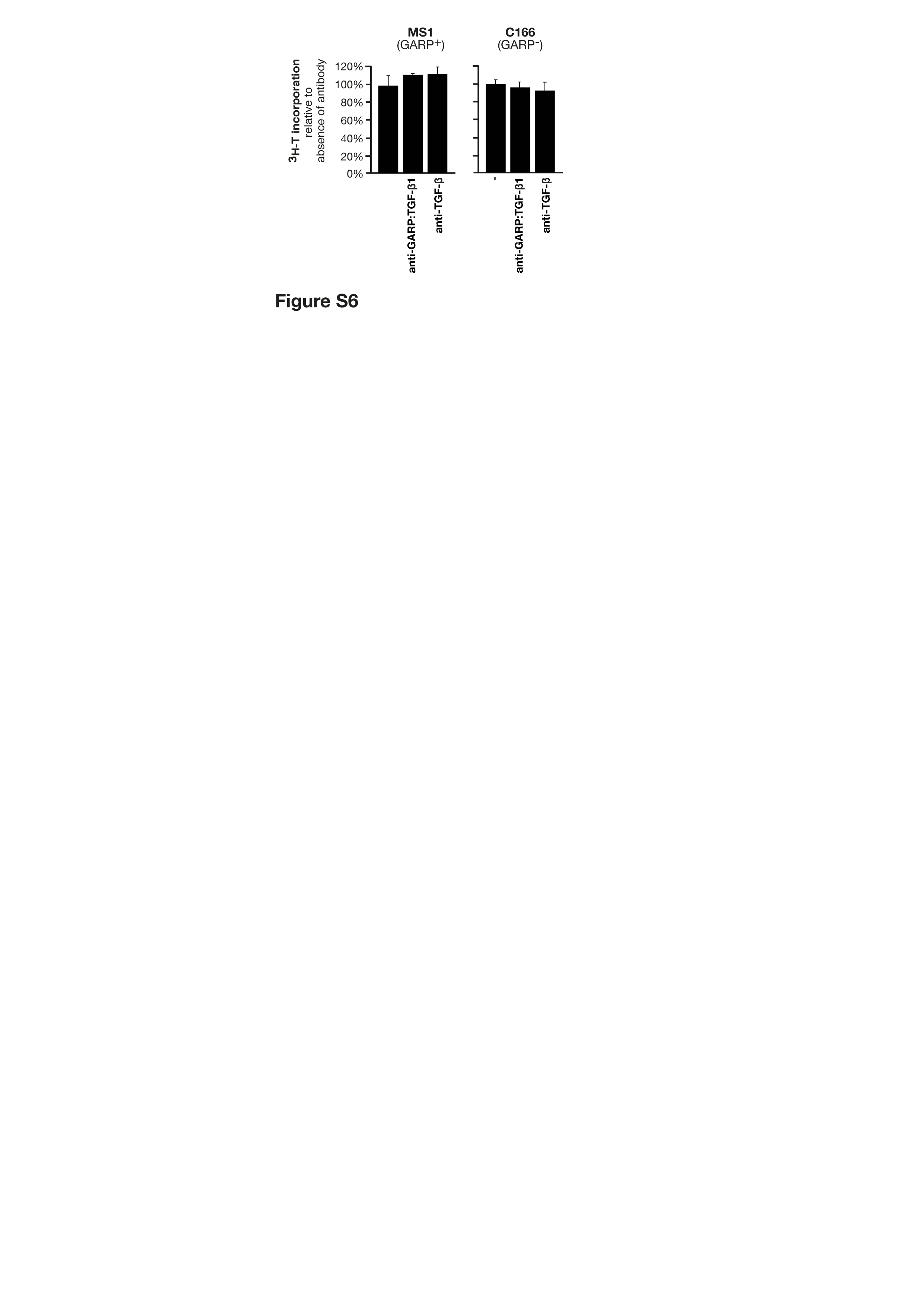

Supplement: Supplementary Figure 6 — C166 and MS1 endothelial cells do not activate TGF-β1 in vitro in absence of stimulus or another cell type. C166 and MS1 cells were used to perform a proliferation assay as described in Figure 5B. Incorporation of 3H-T was measured after 96 hours of culture in the presence of blocking anti-GARP:TGF-β1 (clone 58A2) or neutralizing anti-TGF-β1, β2, β3 (clone 1D11) mAbs. Bars indicate means (+ SD) for triplicate wells. Data representative of 3 independent experiments. [file Image_6.tif]

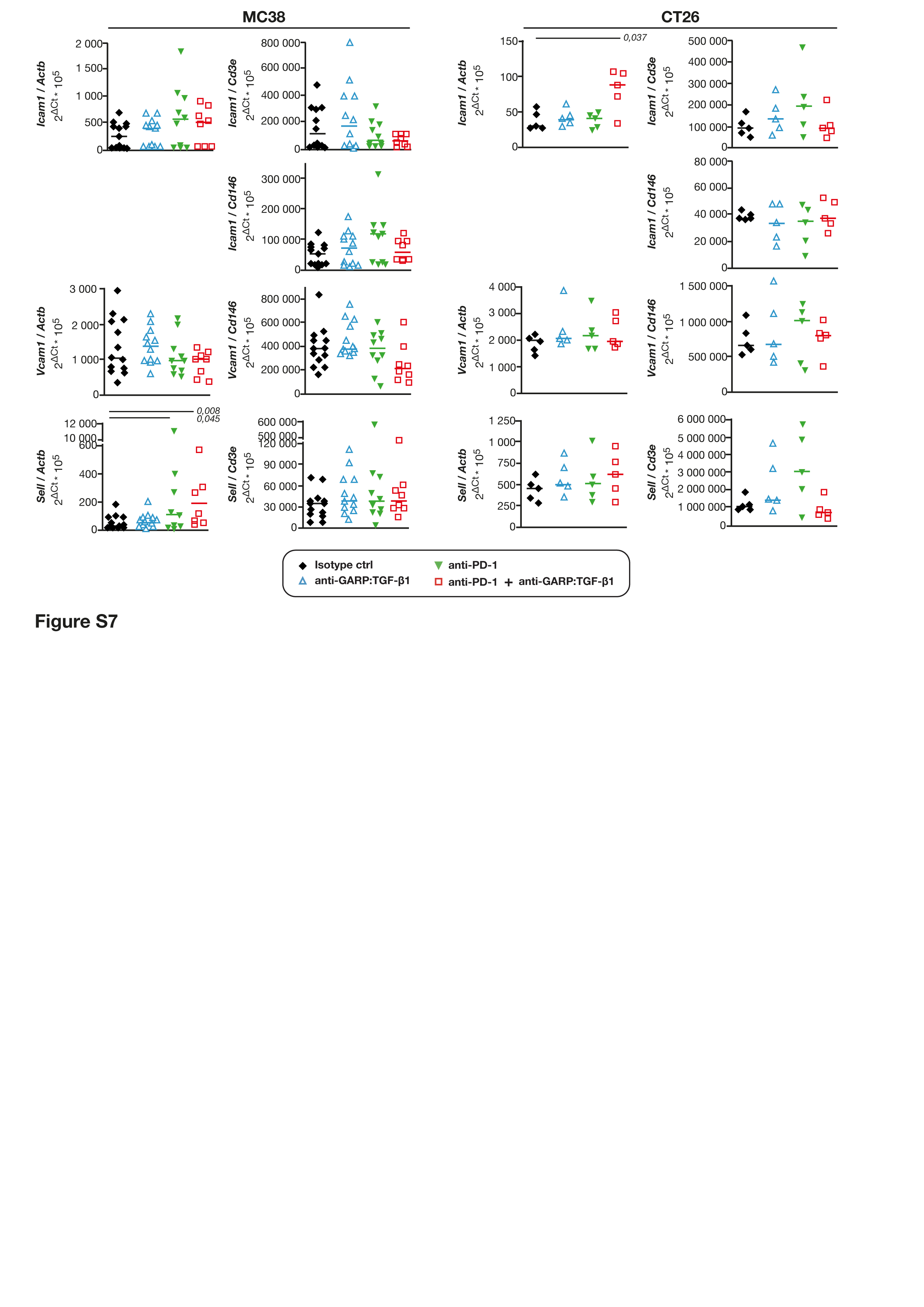

Supplement: Supplementary Figure 7 — Combined GARP:TGF-β1/PD-1 blockade increases expression of Sell but not of Icam1 and Vcam1 in MC38 tumors. RT-qPCR analyses relative to Figure 6A. Graphs show the expression level of Icam1, Vcam1 and Sell normalized by Actb, Cd3e or Cd146. Data points represent values measured in individual mice. Horizontal bars: median per group. Number in italics indicate P values < 0.05 as calculated with a two-sided Wilcoxon test, and numbers in bold indicate fold-changes between groups for selected comparisons. [file Image_7.tif]
